# Evaluating Turkish Readability and Quality of Strabismus-Related Websites

**DOI:** 10.7759/cureus.58603

**Published:** 2024-04-19

**Authors:** Aynur Er, Ibrahim Ethem Ay, Tuğçe Horozoğlu Ceran

**Affiliations:** 1 Ophthalmology, Afyonkarahisar Health Sciences University, Afyonkarahisar, TUR; 2 Ophthalmology, Osmaniye State Hospital, Osmaniye, TUR

**Keywords:** website, strabismus, readability, jama, discern

## Abstract

Background

This cross-sectional study aimed to assess the readability of strabismus-related websites and the quality of their content.

Methodology

This cross-sectional study evaluated the websites on strabismus disease using Ateşman and Bezirci-Yilmaz’s readability formulas, which have been scientifically verified to be effective for Turkish people. The study picked texts from the first 50 websites that appeared on the screen after typing “strabismus treatment” into the Google search engine based on their Turkish reading level and information reliability. In this study, 41 of the first 50 websites were reviewed. Furthermore, two separate senior ophthalmologists evaluated the JAMA and DISCERN indexes, as well as the credibility of the content on these sites.

Results

The Bezirci-Yilmaz readability index indicated that the websites were readable for individuals with an average education level of 10.5 ± 2.3 years. The websites scored an average of 55.2 ± 7.9 on the Ateşman readability formula, indicating that they were readable for students in the 11-12th grade. The websites had an average JAMA score of 0.8 ± 0.7 points and a DISCERN score of 34.2 ± 8.6 points, indicating low-quality content.

Conclusions

The readability of websites providing information regarding strabismus was significantly higher than Turkey’s average educational level. Websites should not only be designed to be easy to read so that strabismus patients may learn about their condition but should also provide higher-quality strabismus content.

## Introduction

Many studies were conducted in the first half of the twentieth century to determine whether written items could be read based on educational levels, which gave rise to the term “readability.” This term refers to the level of education required for an individual to read a text. Initially, many readability formulas were devised to measure the level of education using articles that appeared in newspapers. However, Rudolf Flesch released one of the first significant readability formulas in 1948 [1].

Readability formulas are based on parameters such as how many words a sentence contains and how many syllables each word contains. It is assumed that long sentences composed of long words are more difficult to read.

Patients commonly seek information about their diseases on the internet in clinical practice. Therefore, this study aimed to assess the readability level of strabismus-related websites that strabismus patients are likely to encounter when searching the internet, as well as to assess the quality of content on these sites using the JAMA score [2] and the DISCERN [3] index.

## Materials and methods

This cross-sectional study used Ateşman and Bezirci-Yılmaz’s readability indices, which have been scientifically demonstrated to be effective as the primary formula for Turkish texts [4,5]. In this study, “strabismus treatment” was typed into the Google search engine on May 1, 2022, and the top 50 websites were assessed. The study included 41 websites after removing those that appeared frequently on the search engine because they were shared with advertisements. Websites created by hospitals and comparable health facilities, as well as those published directly by ophthalmologists, were reviewed separately, while newspaper articles were evaluated in a third category.

Websites that opened in other languages during the search, websites with sponsored links and advertisements, duplicate websites, forum sites where detailed information was only visible with a paid membership, and websites not related to the search term were excluded from the study.

The Ateşman readability formula produces a number ranging from 0 to 100 after computations. The higher the score, the more easily the text is understood. In response to the formula’s score, Ateşman published a chart indicating the level of education required to comprehend the text (Table 1). Ateşman readability is calculated using the following formula: Readability score = 198.825 - 40.175 × word length (total syllables/total words) - 2.610 × sentence length (total words/total sentences).

**Table 1:** Ateşman readability formula score and matching education level.

Score	Education level
90–100	It can be read with primary school education of fourth grade and below
80–89	It can be read with education at the fifth or sixth-grade level
70–79	It can be read with education at the seventh or eighth-grade level
60–69	It can be read with education at the ninth or 10th-grade level
50–59	It can be read with education at the 11th or 12th-grade level
40–49	It can be read with education at the 13th to 15th-grade level
30–39	It can be read at the undergraduate level (16th grade)
≤29	It can be read with postgraduate (>16th grade) education

Bezirci-Yılmaz devised a formula that considers the number of words in sentences as well as their syllable count. The result determines the education level at which the content is readable (Table 2).

**Table 2:** Education levels based on Bezirci-Yılmaz readability formula scores/grades.

Score/Class	Education level
1–8	Primary school
9–12	High school
12–16	University
16+	Academic-level education

Additionally, two senior ophthalmologists (A.E. and İ.E.A.), who were blinded, independently assessed the quality of strabismus-related internet information using the JAMA and DISCERN indices.

JAMA index

The JAMA index is used to evaluate information on health-related websites [2]. According to this index, an overall score is obtained by giving 0 or 1 point for authorship, bibliography, patent rights, and topicality (Table 3).

**Table 3:** JAMA scoring system.

Parameters of the JAMA index	Score
Writing	0–1
Source	0–1
Patent right	0–1
Currentness	0–1
Total Score	0–4

DISCERN index

The DISCERN scoring system was developed so that patients, physicians, and online information providers could objectively evaluate the quality of publications on health-related subjects. This index provides a basic grading table for the clarity of the information’s objectives, source identification, balanced and unbiased information, and treatment benefits and risks (Table 4). Each question is assigned a score ranging from 1 to 5. The overall DISCERN score can vary from 16 to 80. A score of 63-80 is deemed excellent, 51-62 good, 39-50 fair, 27-38 poor, and 16-26 very poor.

**Table 4:** DISCERN scoring system.

Questions	Score
Are the objectives clear for the information provided?	1–5
Does the information provided achieve its objectives?	1–5
Is the information provided relevant to the objectives?	1–5
Is it clear which information sources were used for the information provided?	1–5
Is it clear when the information used or reported in the publication was produced?	1–5
Is the information provided balanced and unbiased?	1–5
Does it include details of information sources?	1–5
Does the information provided refer to areas of uncertainty?	1–5
Is it explained how each treatment works?	1–5
Are the benefits of each treatment explained?	1–5
Are the risks and side effects of each treatment explained?	1–5
Is it explained what will happen if no treatment is given?	1–5
Are the effects of treatment options on overall quality of life explained?	1–5
Is it explained that there may be more than one possible treatment option?	1–5
Does the information provided support shared decision-making?	1–5
Evaluate the overall quality of the publication based on the answers to all questions	1–5
Total score	16–80

Data analysis

All collected data were evaluated with SPSS, Version 23.0 (IBM Corp., Armonk, NY, USA). Mean ± standard deviation and median (minimum-maximum) were used for descriptive data. Results were expressed as numbers and percentages for categorical data. Analysis of variance and Kruskal-Wallis tests were performed to evaluate the correlation of categorical variables. Results were accepted at 95% confidence intervals, and p-values <0.05 indicated statistical significance.

## Results

The Bezirci-Yılmaz readability index indicated that webpages could be read by individuals with an average education level of 10.5 ± 2.3 years. Websites were assessed in three independent subgroups based on websites by health organizations, doctors’ internet addresses, and newspapers. There was no statistically significant difference between the readability levels in the subgroup study (p = 0.838) (Table 5).

**Table 5:** Website assessment results based on readability and information quality. ^a^: analysis of variance test;^ b^: Kruskal-Wallis test.

Index	Websites of health organizations (n = 20)	Personal websites of doctors (n = 12)	Newspapers and other similar websites (n = 9)	Total (n = 41)	P-value
Bezirci-Yılmaz (1–16)	10.5 ± 2.4	10.4 ± 1.9	10.8 ± 2.8	10.5 ± 2.3	0.838^a^
Ateşman (0–100)	55.7 ± 8.1	54.9 ± 5.9	54.7 ± 6.3	55.2 ± 7.9	0.681^a^
JAMA (0–4)	1.6 ± 0.5	1.0 ± 0.2	0.5 ± 0.6	0.8 ± 0.7	0.001^b^
DISCERN (16–80)	36.7 ± 6.6	34.6 ± 9.4	24.5 ± 7.9	32.4 ± 8.6	0.080^b^

The websites scored an average of 55.2 ± 7.9 on the Ateşman readability formula, indicating they were readable for 11-12th-grade education. In the subgroup analyses conducted using the Ateşman readability formula, no statistically significant difference in readability was identified between the third set of websites, which included those belonging to health organizations, physicians, and newspaper news (p = 0.681) (Table 5).

The average score for all websites analyzed using the JAMA index was 0.8 ± 0.7. Newspapers and similar websites had much lower quality information (p < 0.001) (Table 5).

The average total score obtained with the DISCERN index was 32.4 ± 8.6. Healthcare websites scored an average of 34.7 ± 6.6 points, but personal websites of doctors had an average of 34.6 ± 9.4 points. Newspapers and other similar media achieved an average score of 24.5 ± 7.9 points (p = 0.08) (Table 5).

## Discussion

This study used the Ateşman readability index and the Bezirci-Yılmaz readability formula, which have been scientifically validated to be reliable for Turkish texts [4,5]. Only a few studies have assessed the readability of Turkish medical writings. Ebem et al. [6] assessed 90 consent forms for intramuscular and intravenous injections using Ateşman and Bezirci-Yılmaz’s readability formulas, revealing low readability. A study indicated that consent forms for surgical interventional procedures in ophthalmology, employing the Ateşman and Bezirci-Yılmaz formulas, were readable with an average of 11 years of education [7]. Further, another study investigated the leaflets of 75 different eye drops for readability in Turkish and discovered that the leaflet forms could be read with an average education of 13 years [8]. In another study on the subject, it was concluded that there is a necessity to make improvements aimed at enhancing the readability of leaflets targeting non-healthcare professionals and addressing users [9].

The American Medical Association conducted one of the reference studies in this field using seven distinct formulas for English, revealing that consent forms obtained from patients before invasive treatments could be read with an average of 15 years of education. The study used seven distinct formulas for English. Given the average level of education in America, it was advised that consent forms be readable by individuals who have completed at least sixth grade [10]. Provided that Turkey’s 2016 average educational level was 6.51 years, important medical documents in Turkey must be readable at the fifth or sixth-grade level [11].

Visitors to websites related to strabismus typically require an educational understanding equivalent to 10.5 ± 2.3 years to comprehend the information presented, which is a level insufficient for accurately informing the general populace. There is scarce research on the readability of Turkish websites. A 2020 study by Çifci et al. [12] investigated the readability of Turkish websites related to substance addiction using Ateşman and Bezirci-Yılmaz readability formulas. The websites were found readable with an average of 14 years of education. Kent et al. [13] assessed the readability of dizziness-related websites using Ateşman and Çetinkaya formulas. The texts were determined to be readable with an average of eight to nine years of education. Panthagani et al. [14] investigated keratoconus-related websites for English readability and found that the content was difficult to read. Furthermore, they assessed the information quality of the websites using JAMA and DISCERN score systems and discovered that the information quality was low. Our findings appear to be congruent with those of Panthagani et al. Laçiner et al. [15] found that websites concerning schizophrenia were readable at 12 years of education level, which is consistent with our findings.

Further, Hutchings et al. [16] evaluated fibroadenoma-related websites for English readability. Determining the readability level in a different language is probably achievable using other readability criteria, but it is difficult to compare one-on-one with our findings. Their findings indicated that half of the websites were difficult to read. We were unable to uncover any convincing data in the literature regarding how frequently strabismus patients utilize the internet to learn about their disease. Nonetheless, as internet use grows in popularity, it would be beneficial to public health if the resources available on the internet were easy to read and of good quality.

The limitation of the study lies in the evaluation of only 41 websites. A more extensive assessment of websites could enhance the comprehensiveness of the study findings. Additionally, the focus on Turkish readability in our article presents a constraint regarding its generalizability. Nevertheless, our findings align with existing literature. It is important to note that despite employing various indices, the evaluations conducted by two independent experts yield subjective data.

## Conclusions

The readability of Turkish websites providing information about strabismus was found to be significantly higher than Turkey’s average education level. Websites should be developed in an easy-to-read manner so that strabismus patients may gain knowledge about their disease. Furthermore, websites on the subject should offer more detailed information concerning strabismus.
